# Influence of [^18^F]FDG-PET/CT on Clinical Management Decisions in Breast Cancer Patients—A PET/CT Registry Study

**DOI:** 10.3390/diagnostics13142420

**Published:** 2023-07-20

**Authors:** Sebastian Werner, Julia Sekler, Brigitte Gückel, Christian la Fougère, Konstantin Nikolaou, Christina Pfannenberg, Heike Preibsch, Tobias Engler, Susann-Cathrin Olthof

**Affiliations:** 1 Department of Diagnostic and Interventional Radiology, University Hospital of Tuebingen, Hoppe-Seyler-Straße 3, 72076 Tuebingen, Germany; julia.sekler@med.uni-tuebingen.de (J.S.); brigitte.gueckel@med.uni-tuebingen.de (B.G.); konstantin.nikolaou@med.uni-tuebingen.de (K.N.); christina.pfannenberg@med.uni-tuebingen.de (C.P.); heike.preibsch@med.uni-tuebingen.de (H.P.); susann-cathrin.olthof@med.uni-tuebingen.de (S.-C.O.); 2 Department of Nuclear Medicine and Clinical Molecular Imaging, University Hospital of Tuebingen, Otfried-Mueller-Straße 14, 72076 Tuebingen, Germany; christian.lafougere@med.uni-tuebingen.de; 3 Cluster of Excellence iFIT (EXC 2180) “Image-Guided and Functionally Instructed Tumor Therapies”, Faculty of Medicine, Eberhard Karls University, 72076 Tuebingen, Germany; tobias.engler@med.uni-tuebingen.de; 4 German Cancer Consortium (DKTK), German Cancer Research Center (DKFZ) Partner Site Tuebingen, 72076 Tuebingen, Germany; 5 Department of Women’s Health, University Hospital Tuebingen, Calwer-Straße 7, 72076 Tuebingen, Germany

**Keywords:** breast neoplasms, positron emission tomography computed tomography, fluorodeoxyglucose F18, survival rate, registries

## Abstract

There is a lack of evidence regarding the clinical impact of [^18^F]fluorodeoxyglucose positron emission tomography/computed tomography ([^18^F]FDG-PET/CT, hereinafter referred to as PET/CT), especially regarding management changes and their link to overall survival. We analyzed 52 PET/CTs in 47 stage I-IV breast cancer patients, selected from a prospective oncological PET/CT registry. Indications for PET/CT were primary staging (*n* = 15), restaging (*n* = 17), and suspected recurrence (*n* = 20). PET/CT-induced management changes were categorized as major or minor. PET/CT-induced management changes in 41 of 52 scans (78.8%; 38 of 47 patients (80.9%)), of which major changes were suggested in 18 of 52 scans (34.6%, 17 of 47 patients, 36.2%). PET/CT downstaged 6 of 15 primary staging patients, excluding distant metastases. Major management changes were documented in 3 of 17 restaging exams. PET/CT ruled out clinically suspected recurrence in 6 of 20 cases and confirmed it in 11 of 20. In three cases, locoregional recurrence had already been diagnosed via biopsy. In 30 of 52 exams, additional diagnostic tests were avoided, of which 13 were invasive. PET/CT-based management changes resulted in a 5-year survival rate of 72.3% for the whole study group, 93.3% for the staging group, 53.8% for the restaging group, and 68.4% for the recurrence group. This study shows that PET/CT significantly impacts clinical management decisions in breast cancer patients in different clinical scenarios, potentially determining the patient’s tumor stage as the basis for further therapy more reliably and by avoiding unnecessary diagnostic tests.

## 1. Introduction

According to the US National Cancer Institute, approximately 13% of women will be diagnosed with breast cancer during their lifetime. For the year 2023, they estimate approximately 298,000 new cases, making up 15.2% of all new cancer cases [1]. Standard local staging in breast cancer comprises mammography and ultrasound. In selected cases, breast MRI is used due to its higher sensitivity for evaluating multifocal and multicentric carcinomas as well as detecting contralateral cancers [2]. The standard systemic imaging modalities for ruling out distant metastases in patients with lymph node metastases (N1) or tumor lesions over 2 cm (T2) are contrast-enhanced CT of the chest and abdomen in combination with bone scintigraphy [3].

The German guidelines recommend [^18^F]fluorodeoxyglucose positron emission tomography/computed tomography ([^18^F]FDG-PET/CT, hereinafter referred to as PET/CT) only in patients with disease recurrence and an unclear status of distant metastases [3]. However, PET/CT can be helpful in several other clinical scenarios. For example, the recent National Comprehensive Cancer Network guideline (Version 3.2022) acknowledges a potential role in detecting regional lymph node involvement and distant metastases in locally advanced breast cancer, including T3 N1 M0 disease [4]. Furthermore, PET/CT improves the evaluation of systemic treatment response compared to computed tomography (CT). It even seems superior to CT and bone scintigraphy in detecting malignancy in patients with suspected recurrent disease [5,6].

However, there is still a lack of evidence regarding the clinical impact of PET/CT, especially regarding management changes and their link to overall survival (OS) and cost-effectiveness. Thus, this prospective study aimed to evaluate changes in clinical management based on PET/CT results and the potential effect on OS in breast cancer patients at a single center.

## 2. Materials and Methods

### 2.1. Study Cohort

This subcohort analysis of our prospective university center PET/CT registry included 47 consecutive in-house patients with breast cancer who underwent 52 PET/CT scans between April 2013 and September 2020. For all included patients, the referring in-house gynecologists filled in a questionnaire regarding their planned therapy before and the performed therapy after PET/CT (for further details, see [7]). After pseudonymization, all data were entered into a good clinical practice–conform registry database supervised by the University Institute of Clinical Epidemiology and Applied Biostatistics. Patients’ follow-up data, primary demographic data, and TNM stagings were extracted from the registry or the in-house clinical patient records. The local institutional review board study reviewed and approved this study (reference number 064/2013BO1).

### 2.2. Indications for PET/CT 

The indication to perform a PET/CT was raised by an interdisciplinary tumor board, based on pre-PET/CT imaging results such as CT, magnetic resonance imaging (MRI), ultrasound, and bone scintigraphy, as well as tumor marker course and clinical data, and grouped as shown in Table 1.

### 2.3. PET/CT Examination Protocol

All examinations were performed with a state-of-the-art clinical PET/CT (Biograph mCT Siemens Healthineers, Knoxville, TN, USA), applying our in-house standard protocol. After at least six hours of fasting, the glucose analogue [^18^F]fluorodeoxyglucose (mean 318.83 ± 26.65 MBq) was injected intravenously and fully diagnostic PET/CT scans from sub-cranial to mid-thigh were acquired 60 min p.i. In 47 of 52 exams, patients received intravenous iodine contrast (mean 115.53 mL ± 8.80 mL; flow rate of 2.5 mL/s; Ultravist 370 Bayer Healthcare Pharmaceuticals Berlin, Germany) and were scanned in the portal venous phase. In 5 patients, the CT scan was acquired without contrast due to contraindications. PET data were corrected for attenuation as well as scatter and reconstructed with three-dimensional ordered-subset expectation maximization (OSEM3D) including time of flight and point spread functions (2 iterations, 21 subsets, Gaussian filter 2 mm). 

### 2.4. Study Analysis

PET/CT-induced clinical management changes between the intended treatment or non-treatment before and after PET/CT were categorized as “major”, “minor”, or “no change”. Changes indicating a major change were as follows: change between treatment and non-treatment strategies, change between treatment modalities (i.e., surgery, radiation, systemic therapy), and a shift from palliative to curative treatment strategies and vice versa. Minor changes were defined as modification within treatment modalities (i.e., change of substance in systemic therapy or change between chemotherapy and immunotherapy) and the avoidance of invasive or non-invasive diagnostic tests. In addition, OS was evaluated for all patients and grouped by indication and management change category.

### 2.5. Statistical Analysis

We performed statistical analysis using GraphPad Prism (GraphPad Prism version 9.3.1 for Windows, GraphPad Software, San Diego, CA, USA, www.graphpad.com, accessed on 17 July 2023) and IBM SPSS Statistics (IBM Corp. Released 2020. IBM SPSS Statistics for Windows, Version 27.0. Armonk, NY: IBM Corp). Continuous data are presented as the mean ± standard deviation (SD). OS rates were calculated as the time interval between PET/CT scan and death and illustrated in Kaplan–Meier graphs. Loss to follow-up or patients who were still alive were considered a censoring event. We regarded a *p*-value < 0.05 as statistically significant. In the case of multiple pairwise comparisons between three different subgroups in the survival analysis, we used Bonferroni correction and adjusted the significance level to *p* = 0.017.

## 3. Results

### 3.1. Study Cohort

The study group included 52 PET/CT scans of 47 breast cancer patients (46 female, mean age 56.9 ± 14.4 years; one male, 65.3 years). The 52 PET/CT examinations comprised multiple scans in 3 patients and single scans in 44 patients. 

The tumor stages of the 47 patients at the time of their initial diagnosis were as follows: stage I, *n =* 10 (21.3%); stage II, *n =* 21 (44.7%); stage III, *n =* 10 (21.3%); stage IV, *n =* 6 (12.8%). Of the 47 patients, 45 had unilateral and 2 had bilateral breast cancer.

The clinical tumor stages (based on the concurrent TNM status determined by all available imaging exams and biopsy results) directly before the 52 PET/CT examinations were the following: no evidence of disease (*n =* 5, 9.6%); stage I (*n =* 1, 1.9%); stage II (*n =* 7, 13.3%); stage III (*n =* 7, 13.3%); stage IV (*n =* 32, 61.5%). Taking the PET/CT results into account the tumor stages changed as follows: no evidence of disease (*n =* 8, 15.4%); stage I (*n =* 4, 7.7%); stage II (*n =* 8, 15.4%); stage III (*n =* 7, 13.5%); stage IV (*n =* 25, 48.1%). Table 2 depicts these numbers stratified by indication subgroup.

The histological subtypes were as follows: no special type (NST), i.e., invasive ductal carcinoma (IDC; *n =* 34, 72.3%); invasive lobular carcinoma (ILC; *n =* 7, 14.9%); invasive medullary carcinoma (*n =* 1, 2.1%); metaplastic carcinoma (*n =* 1, 2.1%) and bilateral (both NST, *n =* 1, 2.1%; NST and metaplastic carcinoma, *n =* 1, 2.1%). In 2 cases (4.3%), the histological subtype could not be derived from the medical records. In the unilateral cases, tumor gradings were as follows: G1 (*n =* 1, 2.1%), G2 (*n =* 25, 53.2%) and G3 (*n =* 18, 38.3%). In the patient with bilateral NST, both tumors were G2. In the second patient with bilateral disease, the NST was G1, and the metaplastic carcinoma was G3. In one patient (2.1%), the grading was not available in the medical records.

**Figure 1:**
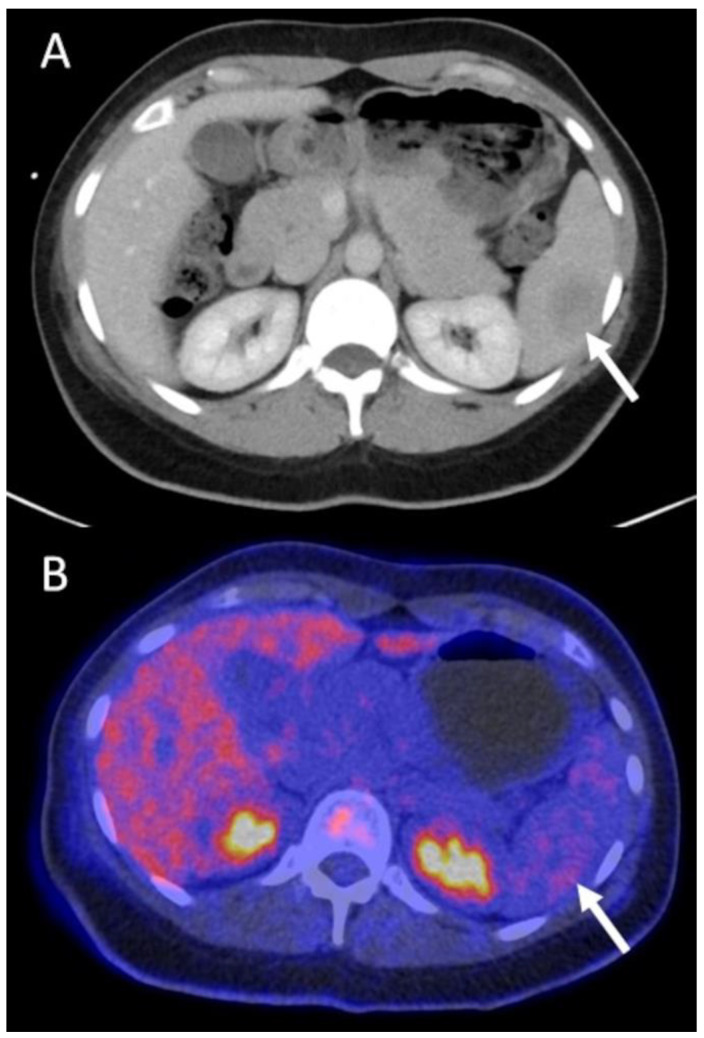
A 37-year old female patient with grade 3 NST of the left breast. Tumor stage pT3, pN1. Initial primary staging CT shows an ill-defined, slightly hypodense splenic lesion, highly suspicious for a distant metastasis (arrow in (**A**)). Additional FDG PET/CT does not show elevated tracer-uptake, excluding a metastasis, thus ruling out M1 stage and leading to a major change in management (arrow in (**B**)). NST = invasive carcinoma of no special type.

**Figure 2:**
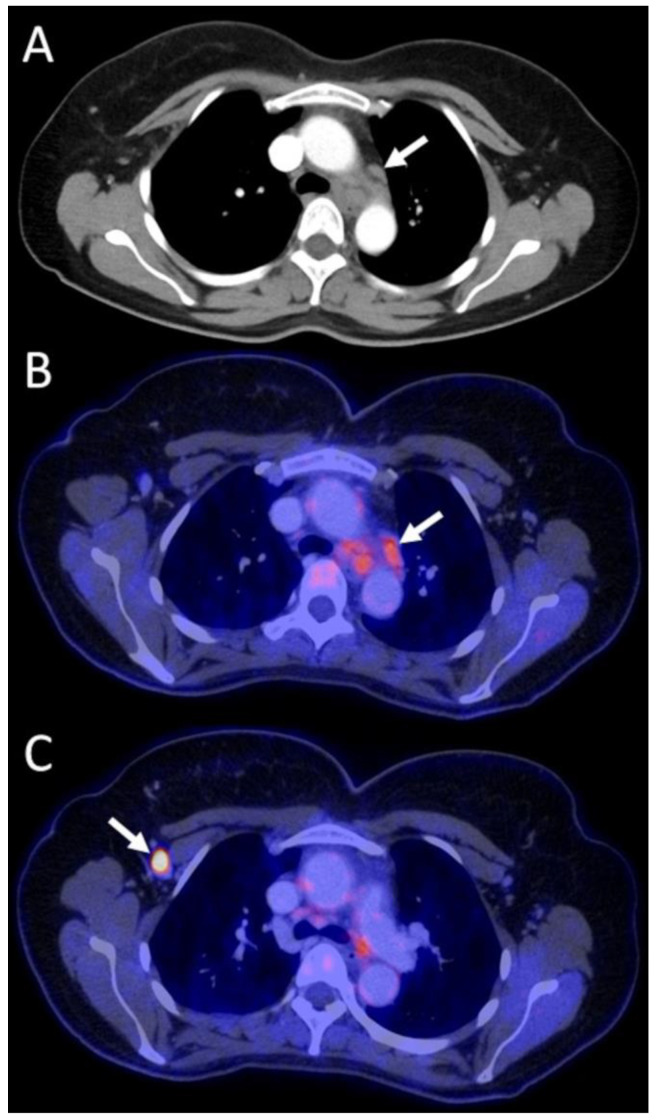
A 44-year old female patient with high-grade NST of the right breast and axillary lymph node metastases. The arrow in the initial contrast-enhanced staging CT shows enlarged mediastinal lymph nodes suspected to be nodal metastases (**A**). In the FDG PET/CT the mediastinal lymph nodes reveal only moderate tracer uptake (arrow, SUVmean 2.5, SUVmax 3.9) (**B**) in comparison to the histopathologically proven axillary lymph node metastases (arrow, SUVmean 8.9, SUVmax 13.2) (**C**). The mediastinal lymph nodes were interpreted as benign, and thus M1 stage was ruled out, allowing inclusion into a clinical trial and modification of systemic therapy. i.e., a major management change. NST = invasive carcinoma of no special type.

**Figure 3:**
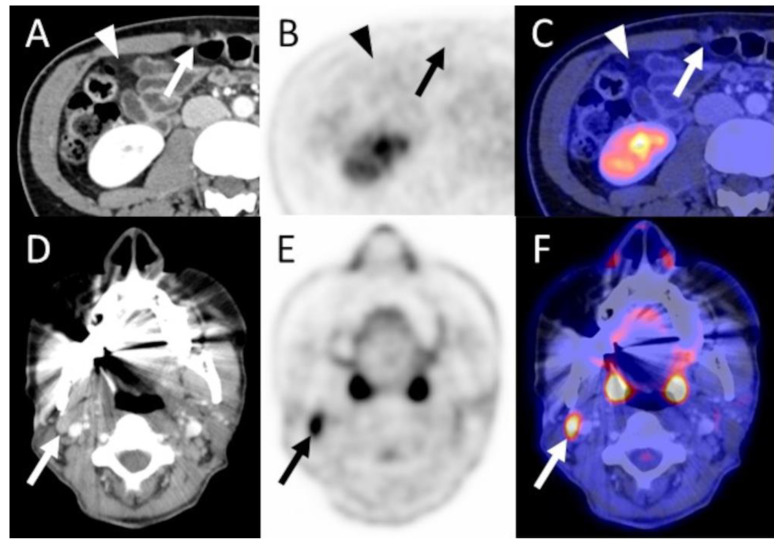
A 42-year old female patient with grade 3 NST in the right breast, stage T1c N0 M0 diagnosed three years prior. FDG PET/CT was performed due to histologically proven axillary nodal recurrence (not shown, SUVmean 5.3, SUVmax 7.4) as well as a nodular peritoneal soft tissue lesion in the right mid-abdomen (arrow in (**A**)) and slight diffuse hyperdense changes to the abdominal fat (arrowhead in (**A**)) detected in the CT, concerning regarding peritoneal metastases. PET/CT did not show pathological tracer uptake of these lesions, and peritoneal metastases were ruled out (arrows and arrowheads in (**B**,**C)**). However, PET/CT allowed for the detection of an additional FDG-avid (SUVmean 5.6, SUVmax 7.8) cervical lymph node (arrows in (**E**,**F**)) which was not easily discernable on CT alone (arrow in (**D**)), due to beam hardening artifacts as well as multiple other slightly enlarged bilateral cervical lymph nodes, and thus was not interpreted as suspicious in the CT. The interdisciplinary tumor board regarded this lymph node as highly suspicious and decided against an additional biopsy. The initially planned resection of the axillary lymph node metastases was not performed. Instead, the patient received systemic treatment. Thus, the FDG PET/CT led to a major change in management. Furthermore, an initially considered diagnostic laparoscopy for evaluation of the suspicious peritoneal CT findings was deemed unnecessary. NST = invasive carcinoma of no special type.

Selected images from three example cases are presented in Figure 1, Figure 2 and Figure 3. 

### 3.2. Management Changes after PET/CT 

Of the 52 PET/CT exams, 41 (78.8%), which were performed in 38 of the 47 patients (80.9%), led to a change in management, of which 18 were major (34.6%) and 23 (44.2%) were minor.

In 11 cases (21.2%), the exam did not lead to a management change. In 13 of 52 cases, invasive diagnostic tests could be dispensed with. In 17 of 52 additional cases, non-specified or non-invasive diagnostic tests were avoided. Table 3 depicts further details.

In the primary staging group, PET/CT led to a downstaging in 6 of 15 patients by excluding distant metastases and, thus, a change from a palliative to a curative therapy approach, classified as major changes. In 4 of 15 patients, PET/CT avoided unnecessary biopsies, classified as minor management changes. 

In the restaging group (*n =* 17), the three major management changes encompass the initiation of systemic therapy (*n =* 1) and vice versa (*n =* 1) and the extension to additional necessary radiotherapy (*n =* 1). Minor changes encompassed the modification of systemic therapies (8 of 17) and the avoidance of unnecessary biopsies (3 of 17). 

The major management changes in the recurrence group (*n =* 9 of 20) included a shift from non-treatment to treatment (*n =* 2) and vice versa (*n =* 1), as well as fundamental therapy changes (*n =* 6; for details, see Table 2). Minor changes included avoiding further biopsies (6 of 20) and modifications in systemic therapy (2 of 20). 

### 3.3. Survival Analysis

**Figure 4:**
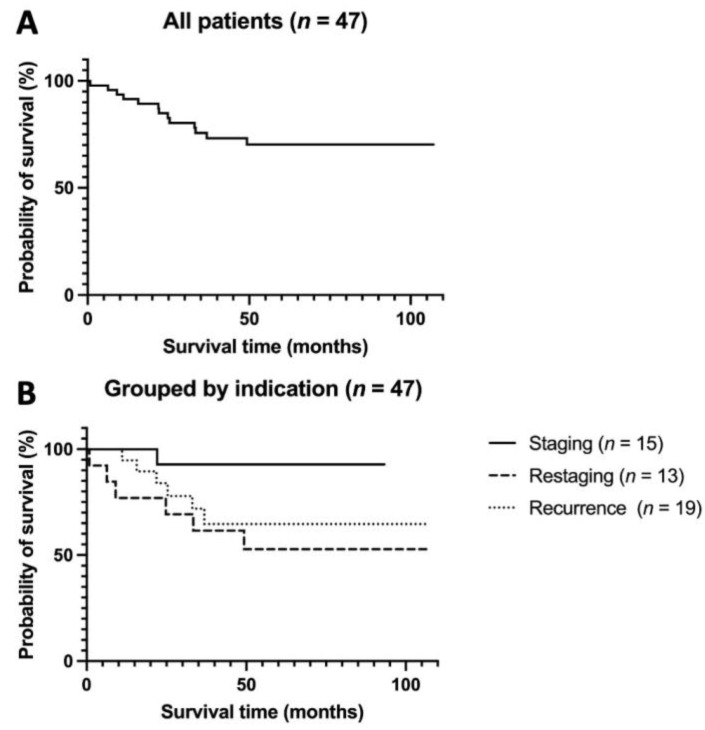
Kaplan–Meier OS analysis for (**A**) all patients and (**B**) grouped by indication subgroup. (**A**) Kaplan–Meier OS analysis of all 47 patients. Five-year OS was 72.3% (mean 82.2 months, 95% CI 70.7–93.7). (**B**) Kaplan–Meier OS analysis of 47 patients according to the three indication subgroups. Five-year OS was 93.3% in the staging group (mean 88.2 months, 95% CI 78.6–97.8), 53.8% in the restaging group (mean 66.6 months, 95% CI 42.2–90.9, *p* = 0.069), and 68.4% in the recurrence group (mean 77.9 months, 95% CI 59.0–96.8). OS = overall survival.

The 5-year OS for our whole study group of all 47 patients was 72.3% (mean 82.2 months; 95% confidence interval (CI) 70.7–93.7; see Figure 4A).

Grouped by indication, 5-year OS was highest in the staging group with 93.3% (mean 88.2 months; 95% CI 78.6–97.8; with curative treatment in 13 of 15 patients) and lowest in the restaging group with 53.8% (mean 66.6 months; 95% CI 42.2–90.9, *p* = 0.069; with 11 of 13 palliatively treated patients, of which 8 had progressive disease). The recurrence group lay in between with a 5-year OS of 68.4% (mean 77.9 months, 95% CI 59.0–96.8; with 8 of 19 curative and 11 of 19 palliative patients). However, the differences in OS between these groups were statistically insignificant (*p* = 0.073, see Figure 4B).

**Figure 5:**
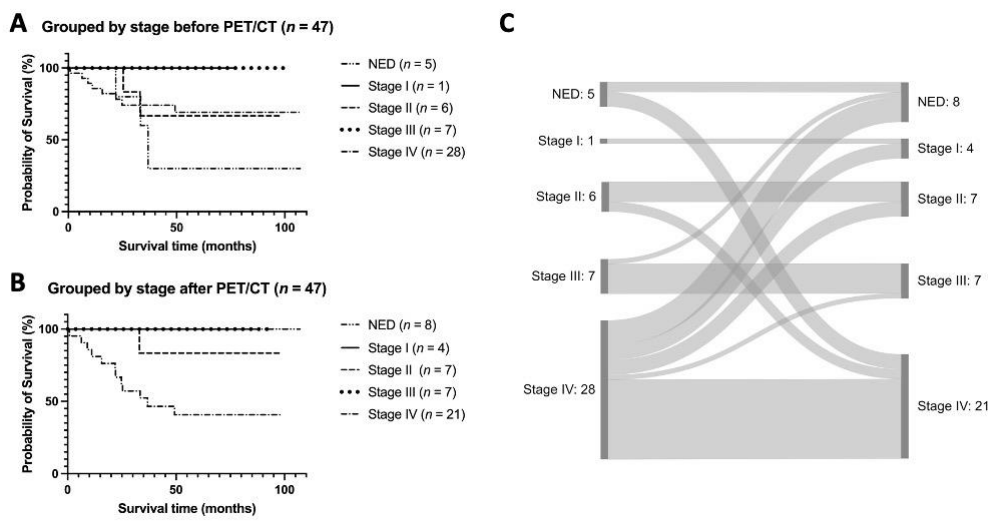
Comparison of OS according to the patients’ tumor stages (**A**) before and (**B**) after FDG PET/CT, as well as (**C**) illustration of the stage shifts. (**A**) Kaplan–Meier OS analysis of 47 patients according to the tumor stage before PET/CT. Five-year OS was lowest in patients with NED with 40%. Five-year OS was 71.4% in stage IV patients, 100% in stage III patients, 66.6% in stage II patients, and 100% in stage I patient. (**B**) Kaplan–Meier OS analysis of 47 patients according to the tumor stage after PET/CT. Five-year OS was lowest for stage IV patients with 42.8%. Five-year OS was 100% in stage III patients, 85.7% in stage II and 100% in stage I patients and NED. (**C**) Sankey diagram illustrating the migration of patients between tumor stages before and after PET/CT. OS = overall survival; NED = no evidence of disease.

We also analyzed and compared survival rates stratified by the patients’ tumor stage before and after PET/CT. The results, as well as a Sankey diagram illustrating the patients’ migration between tumor stages before and after PET/CT, are presented in Figure 5.

### 3.4. Assessment of Potential Patient Benefit 

#### 3.4.1. Primary Staging Patients (*n =* 15/52)

PET/CT is recommended for primary staging, mainly in patients with advanced tumors in stage III [8]. However, our study shows that PET/CT helps to clarify uncertain conventional imaging findings, independent of the initial breast tumor size with seven T1, four T2, two T3 and two T4 tumors. This has also been demonstrated in a survey of 232 triple-negative breast cancer patients, in which PET/CT detected otherwise occult metastases in 5% of patients in stage IIa and 15% of patients in stage IIB [9]. Furthermore, PET/CT excluded suspicious metastatic lesions in 6 of 15 patients, leading to a downstaging with a change from a palliative to a curative therapy approach. Patients profited from this stage-appropriate therapy associated with superior survival data [10,11]. In another 4 of 15 patients, PET/CT avoided unnecessary biopsies associated with high psychological stress, potentially influencing cancer progression [12,13].

#### 3.4.2. Restaging Patients (*n* = 17/52)

Our restaging group showed a high number of therapeutic management changes after PET/CT (*n* = 14/17 exams, 3 of which being major changes). The OS in this group was the lowest (median 66.6 months; 95% CI 42.2–90.9, *p* = 0.069). Even though PET/CT allowed modifications of the systemic therapy based on the more precise assignment of the tumor stage, we could not prove a superior survival of our patients, due to the systemic character of the treatment.

#### 3.4.3. Recurrence Patients (*n =* 20/52)

This group showed management changes in 17 of 20 exams, of which 9 were major and 8 minor. PET/CT could rule out recurrence in 6 cases in which conventional imaging (*n* = 5) or clinical symptoms (*n* = 1) had raised suspicion. Consequently, those patients did not receive any unnecessary systemic therapy associated with potential side effects [14]. On the other hand, in 11 cases with unclear or unremarkable conventional imaging results, PET/CT confirmed the recurrence, initiating an immediate systemic therapy start. In the remaining 3 cases with histologically proven locoregional recurrence, PET/CT either ruled out distant metastases (*n* = 2) or proved them (*n* = 1), affecting subsequent therapies.

## 4. Discussion

Our study shows that PET/CT strongly impacts the therapy given to breast cancer patients in multiple clinical scenarios, leading to therapeutic management changes in 79% of cases. In 35% of cases, these were major therapy changes.

In our primary staging group, PET/CT induced 66.6% modifications, including minor and major management changes. However, as clinical outcomes depend on the patient’s age, tumor stage and grade and type of therapy, those issues contribute to heterogeneous patient subgroups. Thus, it is difficult to directly translate the PET/CT-induced management changes into improved clinical outcomes, as also mentioned by Han et al. [15]. In their meta-analysis of 4276 stage I–IV breast cancer patients, therapeutic approaches were altered based on FDG PET imaging in approximately 20% of patients. Regarding the primary staging in 103 high-risk breast cancer patients, Vogsen et al. noticed PET/CT-induced treatment changes in 39%. Thereafter, surgical treatment was changed in 23% due to the detection of metastatic disease, systemic therapy was changed in 25%, and radiotherapy was altered in 38% [16]. Gunalp et al. retrospectively examined 336 PET/CT examinations for initial staging in 141 pre- and 195 post-operative breast cancer patients. In pre-operative patients, PET/CT changed the staging with an impact on therapeutic management in 47% due to the detection of extra-axillary and distant metastases. Of these, 15% (3/19 patients) in stage I, 25% (13 of 51) in stage IIA, 48% (24 of 49) in stage IIB, 58% (7 of 12) in stage IIIA and 100% (2 of 2) in stage IIIB) [17]. This study emphasizes our findings, namely that PET/CT for primary staging can be critical even in the early stages I and II, although the guidelines recommend PET/CT mainly in patients with advanced tumors in stage III [8]. Moreover, in post-operative patients, additional PET/CT findings changed radiotherapy planning in 22 (11%) patients, and chemotherapy was adapted to metastatic disease in 24 (12%) patients [17]

Interestingly, our results regarding the stage migration with 0 of 15 up- and 6 of 15 downstagings were different compared to a study by Vogsen et al. [16], with 40 of 103 (39%) upstagings, due to either distant or lymph node metastatic disease in PET/CT compared to cTNM staging by conventional mammography with or without MRI. One reason for this discrepancy with the literature might be that unclear conventional imaging findings were the main indication for primary staging with PET/CT in our study cohort.

PET/CT plays a critical role in the response assessment of systemic therapies in metastatic breast cancer, as 14 of 17 of our restaging patients underwent PET/CT-induced management changes. This is in line with a study of 300 metastatic breast carcinoma patients, in whom PET/CT detected progressive disease five months earlier than conventional imaging, resulting in higher survival data in patients with PET/CT compared to patients solely examined via CT [18]. PET/CT is superior to conventional imaging in the detection of distant metastases, especially in bone metastases, enabling early response assessment even after three cycles of systemic therapy, as a study by Ulaner et al. [6] showed for the evaluation of bone metastases. Thus, patients with a response can benefit from an early therapy evaluation, both in terms of a reduction of psychological strain of unclear therapy success and a superior prediction of OS compared to conventional imaging [19]. In contrast, patients with progressive disease benefit from early detection shifting to a different systemic therapy or different treatment. 

In our 17 patients with suspected, yet not proven recurrence, the high rate of 14 positive results is similar to the literature. Liu et al. found PET-positive results in 28 of 30 patients with asymptomatic tumor marker increase and negative or equivocal conventional local and whole body imaging results, of which 14 were local recurrences, 12 axillary lymph node recurrences, and 9 distant recurrent lesions [20]. However, the recurrent lesions’ location differs from our study. We found distant metastases in 5 cases, nodal metastases in 4 cases, and nodal and distant metastases in 3 cases. In another study with 228 asymptomatic patients with rising tumor markers, PET/CT scans were positive in 181 patients (79.5%), with 187 true diagnosed recurrences and 123 treatment modifications (54%) [21]. And finally, the meta-analysis of 1752 patients by Xiao et al. [22] also demonstrated PET/CT’s value in the diagnosis of disease recurrence with a pooled sensitivity of 90% and specificity of 81%. These findings underline the fundamental role of PET/CT in the early and correct identification of recurrences and their associated therapeutic changes, which are essential for patients’ prognosis.

Our data show that with the help of PET/CT, it is possible to classify patients more reliably into exact tumor stages, which is the basis for further therapy. Thus, in 5 of 28 patients initially recorded as stage IV, no metastases could be detected via PET/CT, sparing these patients unnecessary systemic therapy. In contrast, in 3 of 5 patients in which initially no metastases had been detected, PET/CT detected distant metastases, facilitating early therapy initiation. Furthermore, the exact staging of patients via PET/CT is reflected in the comparison of survival data before and after PET/CT.

Since we based this study on registry data, it comes with the typical limitations of a registry study, such as patient selection bias [23]. The validity of our results is somewhat limited by the fact that our study cohort was relatively small and furthermore performed subgroup analyses. Moreover, we only included in-house patients to obtain representative follow-up data from a single center. However, the decision to perform PET/CT was made by an interdisciplinary tumor board, according to strict and uniform clinical criteria, representing the daily clinical routine. Additionally, our results are dependent on the quality of the completed questionnaires of the referring physicians. Lastly, real proof of benefits from PET/CT is only valid via a randomized controlled trial, including follow-up data investigating the correctness of the PET/CT report. However, performing a randomized controlled trial seems unethical due to the already widespread utilization of PET/CT imaging with its proven higher diagnostic accuracy compared to conventional imaging.

## 5. Conclusions

Despite these limitations, this study’s registry data can be considered a valuable means of producing practice-based evidence for the effectiveness of PET/CT as a diagnostic test in breast cancer patients in the daily clinical routine, especially for primary staging as well as recurrence patients.

## Figures and Tables

**Table 1:** Indications for the 52 PET/CT examinations in the study cohort.

	Subcategory	Reason for Restaging/Suspected Recurrence before PET/CT	*n*
Group A Primary staging *n =* 15 (28.8%)	Equivocal/suspicious findings in conventional imaging examinations		14
	Clinical trial		1
Group B Restaging *n* = 17 (32.7%)	Assessment of therapy response	clinical	1
		clinical + laboratory	1
		clinical + laboratory + imaging	2
		laboratory	2
		imaging	7
	Assessment of residual tumour (vitality)	imaging	3
	Follow-up after high-risk CA and non-optimal treatment	-	1
Group C Recurrence *n* = 20 (38.5%)	Suspected recurrence	clinical	2
		clinical + imaging	1
		clinical + laboratory + imaging	2
		laboratory	1
		laboratory + imaging	1
		imaging	9
		incidental findings during cholecystectomy	1
	Proven locoregional recurrence	exclusion of distant metastases	3
Total *n* = 52 (100%)			

**Table 2:** Clinical tumor stage directly before and after the 52 PET/CT examinations, based on concurrent TNM status. TNM status before PET/CT is determined via all available imaging exams and biopsy results. TNM status after PET/CT takes the PET/CT result into account.

Indication	Stage	Before PET/CT	After PET/CT
Staging (*n =* 15 exams)	1	1 (6.7)	3 (20.0)
	2	2 (13.3)	5 (33.3)
	3	4 (26.7)	5 (33.3)
	4	8 (53.3)	2 (13.3)
Restaging (*n =* 17 exams)	NED	2 (11.8)	2 (11.8)
	1	0 (0.0)	0 (0.0)
	2	0 (0.0)	0 (0.0)
	3	2 (11.8)	1 (5.9)
	4	13 (76.5)	14 (82.4)
Recurrence (*n =* 20 exams)	NED	3 (15.0)	6 (30.0)
	1	0 (0.0)	1 (5.0)
	2	5 (25.0)	3 (15.0)
	3	1 (5.0)	1 (5.0)
	4	11 (55.0)	9 (45.0)

Numbers in parentheses indicate %; NED = no evidence of disease.

**Table 3:** Overview of the FDG PET/CT-induced management changes in 47 breast cancer patients with 52 FDG PET/CT scans stratified by three different indication groups.

	Initial Stage at Primary Breast Cancer Diagnosis	Management Strategy		Curative/Palliative Change						
Indication for PET/CT		Pre PET/CT	Post PET/CT	Pre PET/CT	Post PET/CT	PET/CT Result	Avoidance of Additional Diagnostics	No. of Scans	Grading of Management Changes	Potential Benefit of PET/CT--Induced Management Changes
Group A Staging (*n =* 15 scans in 15 patients)	Stage I: *n =* 3 Stage II: *n =* 6 Stage III: *n =* 5 Stage IV: *n =* 1	surgery + systemic	surgery + systemic	P	C	downstaging, IV → I	yes, invasive	1	Major (*n =* 6)	better customized therapy, major psychological benefit of downstaging, avoidance of invasive tests
						downstaging, IV → IIa		1		
		surgery + systemic + RTX	surgery + systemic + RTX	P	C	downstaging, IV → IIb	no	2		better customized therapy, major psychological benefit of downstaging
		systemic	systemic	P	C	downstaging, IV → IIIa	yes, invasive	1		better customized therapy, major psychological benefit of downstaging, avoidance of invasive tests
		systemic + RTX	systemic + RTX	P	C	downstaging, IV → I	yes, invasive	1		better customized therapy, major psychological benefit of downstaging, avoidance of invasive tests
		surgery + systemic + RTX	surgery + systemic + RTX	C	C	no change in stage	yes, not specified	4	Minor (*n =* 4)	avoidance of additional tests
		surgery + systemic + RTX	surgery + systemic + RTX	C	C	no change in stage	no	3	No change (*n =* 5)	
		systemic + RTX	systemic + RTX	P	P	no change in stage	no	2		
Group B Restaging (*n =* 17 scans in 13 patients)	Stage I: *n =* 3 Stage II: *n =* 5 Stage III: *n =* 2 Stage IV: *n =* 3	non-treatment	systemic	C	P	progressive disease	yes, not specified	1	Major (*n =* 3)	earlier therapy start with improved outcome, avoidance of additional tests
		systemic	non-treatment	P	P	complete remission	no	1		ending of therapy and preventing its side effects
		systemic	systemic + RTX	P	P	progressive disease	no	1		earlier therapy start with improved outcome
		systemic	systemic (modified)	P	P	progressive disease	yes, invasive	2	Minor (*n =* 11)	better customized therapy/early replacement of ineffective therapy, avoidance of invasive tests
							yes, not specified	1		better customized therapy/early replacement of ineffective therapy, avoidance of additional tests
							no	5		better customized therapy/early replacement of ineffective therapy
		surgery + systemic	surgery + systemic	C	C	partial remission	yes, invasive	1		avoidance of invasive tests
		systemic	systemic	P	P	stable disease	yes, invasive	1		avoidance of invasive tests
						mixed	yes, not specified	1		avoidance of additional tests
		non-treatment	non-treatment	C	C	complete remission	no	1	No change (*n =* 3)	
		systemic	systemic	P	P	progressive disease	no	1		
						partial remission		1		
Group C Recurrence (*n =* 20 scans in 19 patients)	Stage I: *n =* 4 Stage II: *n =* 10 Stage III: *n =* 3 Stage IV: *n =* 2	non-treatment	systemic	C	P	recurrence	yes, invasive	1	Major (*n =* 9)	earlier therapy start with improved outcome, avoidance of invasive tests
						recurrence	yes, not specified	1		earlier therapy start with improved outcome, avoidance of invasive tests
		surgery	systemic	C	P	recurrence	yes, not specified	1		better customized therapy, avoidance of additional tests
				P	P		yes, invasive	1		better customized therapy, avoidance of invasive tests
		systemic	surgery + systemic	C	C	recurrence	yes, not specified	1		earlier therapy start with improved outcome, avoidance of additional tests
				C	C		no	1		earlier therapy start with improved outcome
				P	P		no	1		earlier therapy start with improved outcome
		systemic + RTX	non-treatment	P	C	no recurrence	yes, invasive	1		avoidance of unnecessary therapy and its side effects, avoidance of invasive tests
		systemic + RTX	surgery + systemic	P	C	recurrence	yes, not specified	1		better customized therapy, avoidance of additional tests
		systemic	systemic (modified)	P	P	recurrence	yes, not specified	1	Minor (*n =* 8)	better customized therapy, avoidance of additional tests
							yes, invasive	1		better customized therapy, avoidance of invasive tests
		non-treatment	non-treatment	C	C	no recurrence	yes not specified	3		avoidance of additional tests
							yes, invasive	1		avoidance of invasive tests
		systemic	systemic	P	P	recurrence	yes, not specified	1		avoidance of additional tests
							yes, non-invasive	1		avoidance of additional tests
		systemic	systemic	C	C	no recurrence	no	1	No change (*n =* 3)	
		systemic	systemic	P	P	recurrence	no	2		

P = palliative; C = curative; PET/CT = [^18^F]fluorodeoxyglucose positron emission tomography/computed tomography; RTX = radiotherapy.

## Data Availability

The data presented in this study are available in the Supplementary Material (survival_data.xlsx; supplementary_table.xlsx).

## Supplementary Materials

The following supporting information can be downloaded at: https://www.mdpi.com/article/10.3390/diagnostics13142420/s1, Table S1: Supplementary Table; Table S2: Survival Data.
